# Pneumocephalus: A Rare Complication of Sinusitis in a Pediatric Patient

**DOI:** 10.7759/cureus.65370

**Published:** 2024-07-25

**Authors:** Emily C Barbee, Jeremy Boroff

**Affiliations:** 1 Emergency Medicine, Mercy Health - St. Rita's Medical Center, Lima, USA

**Keywords:** atraumatic pneumocephalus, cribriform plate, valsalva maneuver, sinusitis, pediatric, spontaneous pneumocephalus

## Abstract

Pneumocephalus is uncommon, mostly arising as a result of surgery, trauma, infection, or neoplasm. Spontaneous occurrence is extremely rare and few case studies have been published. Pneumocephalus may also present as a complication of sinusitis and is a potential emergency. It is necessary to make a prompt diagnosis in order to direct treatment toward the underlying cause. Although usually asymptomatic, pneumocephalus can lead to tension pneumocephalus or septic meningitis as the result of infection from bacteria. We present a case of spontaneous, non-traumatic pneumocephalus in the setting of pansinusitis in a pediatric patient. Our aim is to briefly discuss the etiology and emergency department evaluation and management of patients with pneumocephalus.

## Introduction

Pneumocephalus is characterized by the pathological collection of gas within the cranial cavity. Pneumocephalus was first described in 1741 by Lecat et al. [1]. The main cause of pneumocephalus is head trauma, which is responsible for up to 74% of all cases. Other causes of pneumocephalus include intracranial neoplasms, infections, neurosurgery, paranasal sinus surgery, and diagnostic or neurosurgical interventions such as lumbar puncture [2]. However, in rare cases, pneumocephalus can occur spontaneously. Spontaneous non-traumatic occurrence represents only 0.6% of all cases and was first described in 1954 by Jelsma and Moore [1]. Few case studies have been published to date. The most common causes of spontaneous, non-traumatic pneumocephalus include otogenic pneumocephalus, bone defects, malformations (birth defects), infections, and tumors [3]. In our review of the literature, only three previous pediatric cases of pneumocephalus in the setting of sinusitis have been described [4,5]. Here, we present the case of a pediatric patient with spontaneous, non-traumatic pneumocephalus in the setting of pansinusitis and provide a review of the diagnosis and management of pneumocephalus.

## Case presentation

An 11-year-old female patient with a past medical history of headaches and sinusitis presented with the complaint of a frontal headache and nasal congestion for three days. The patient had been blowing her nose frequently and reported dizziness with position changes. The patient had reported a fever of 101 degrees Fahrenheit, one episode of non-bilious, non-bloody vomiting, and swelling of the left eye on the day prior to presentation. The patient’s parent had become concerned because the patient developed drooping of the left eyelid on the day of presentation. She had been complaining of bilateral hand weakness and had had difficulty opening water bottles for the past few days. Parents denied head trauma, loss of consciousness, ataxia, or seizure activity. The patient denied cough, sore throat, rash, myalgias, numbness, tingling, vision changes, hearing changes, abdominal pain, or chest pain. The patient denied recent sick contacts or travel. Parents reported no recent surgery, intravenous (IV) placement, or other procedures. Of note, the patient had multiple visits to her primary care physician's office for headaches in the 3 months before presentation. The documentation of these previous office visits described headaches associated with photophobia and was severe enough that the patient had missed school. She had been diagnosed at the primary care office with sinusitis three months prior to her ED presentation and treated with amoxicillin-clavulanate. The day before the presentation, her primary care office again diagnosed her with sinusitis and prescribed azithromycin and prednisone.

At the time of presentation, the patient’s temperature was 101.6 degrees Fahrenheit. Her vital signs were otherwise normal. Examination revealed an alert, cooperative child. She had no rash, lymphadenopathy, or evidence of external traumatic injury. Ear, nose, throat, and eye exam revealed subtle drooping of the left upper eyelid. The vision was normal. No nasal discharge was noted. The heart, lung, and abdominal exams were within normal limits. Examination of the neck revealed full range of motion and absence of meningismus. Her Glasgow Coma Scale (GCS) was 15. Neurologic examination revealed no other focal deficit. The patient received a 1-liter normal saline bolus, ketorolac 15 mg IV, metoclopramide 5 mg IV, and diphenhydramine 12.5 mg IV for headache. A CBC with differential, BMP, anion gap, and serum osmolality were obtained and were unremarkable. COVID-19 and influenza swabs and serum pregnancy tests were negative. A C-reactive protein (CRP) level was elevated at 13.13 mg/dL (Table 1).

**Table 1:** Relevant laboratory results on initial presentation.

Laboratory tests	Results	Reference range & Units
WBC	9.1	4.8-10.8 X 10^3^ /mm^3^
Seg neutrophils	78.3	55-70%
Lymphocytes	11.1	20-40%
Monocytes	9.8	2-8%
Eosinophils	0.2	1-4%
Basophils	0.3	0.5-1%
C-reactive protein	13.13	0.00-1.00 mg/dL

**Figure 1:**
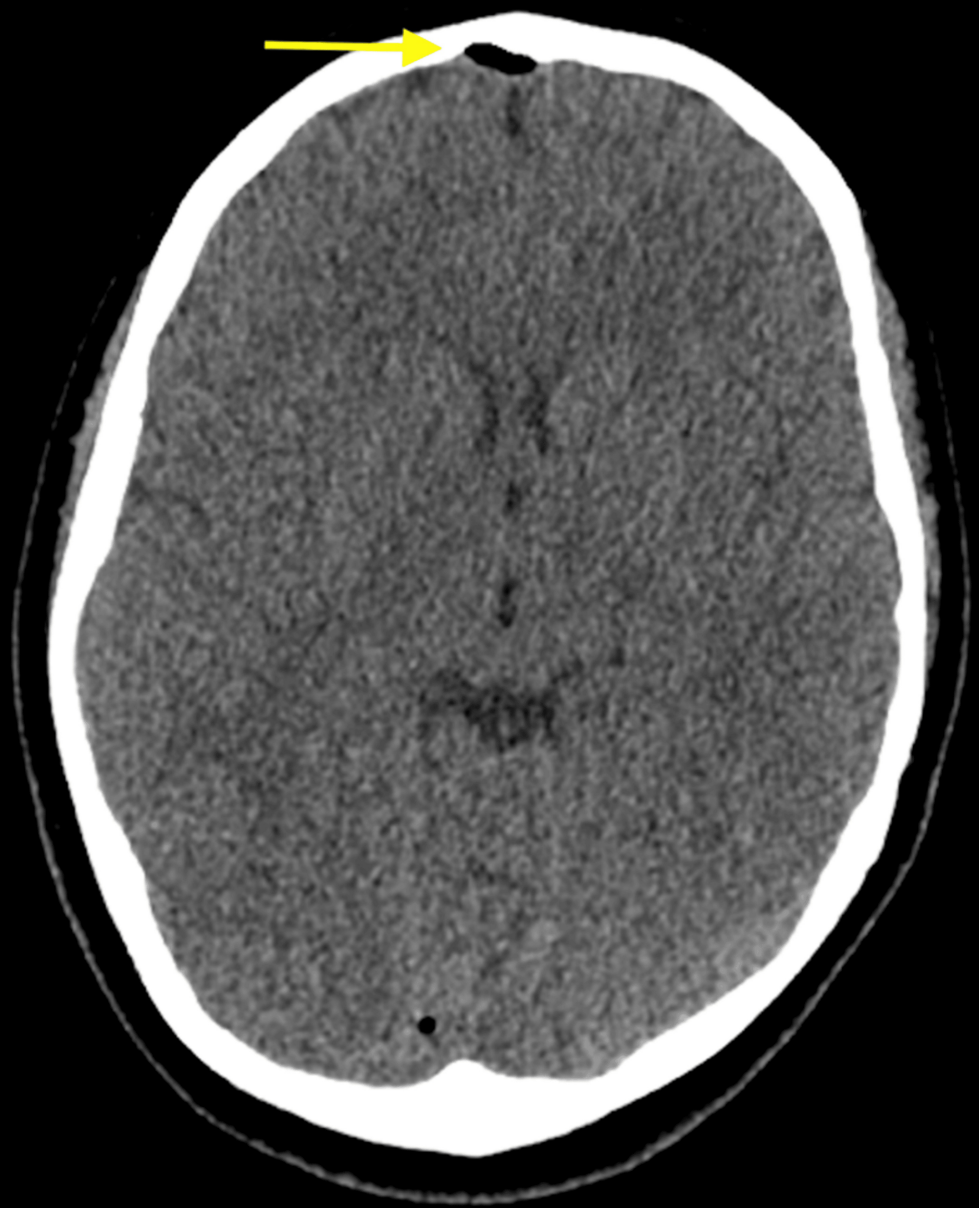
Unenhanced axial computed tomography image of the brain demonstrates air present within the extra-axial space along the frontal lobe, slightly to the right of the midline.

**Figure 2:**
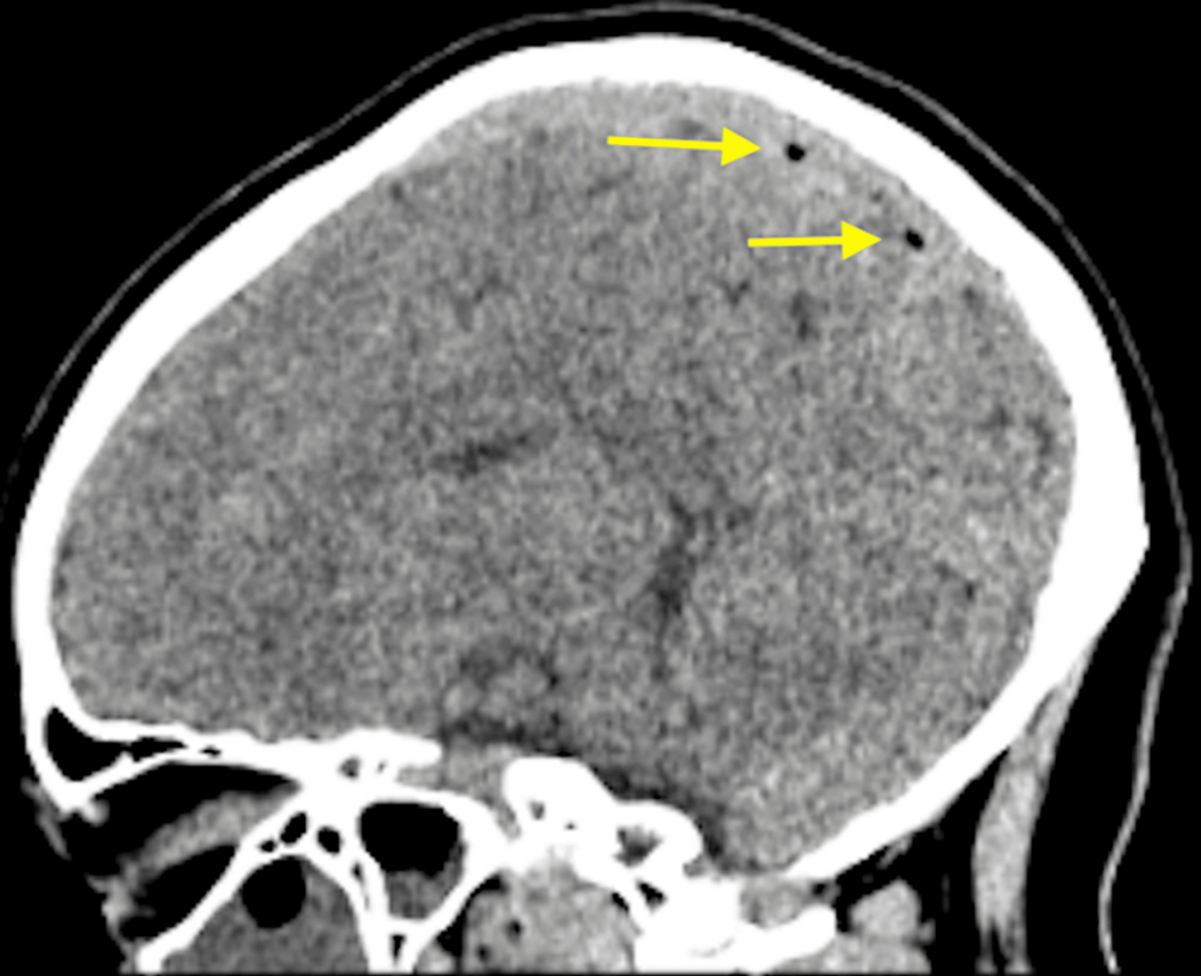
Unenhanced sagittal computed tomography image of the brain demonstrates air present within the superior sagittal sinus.

**Figure 3:**
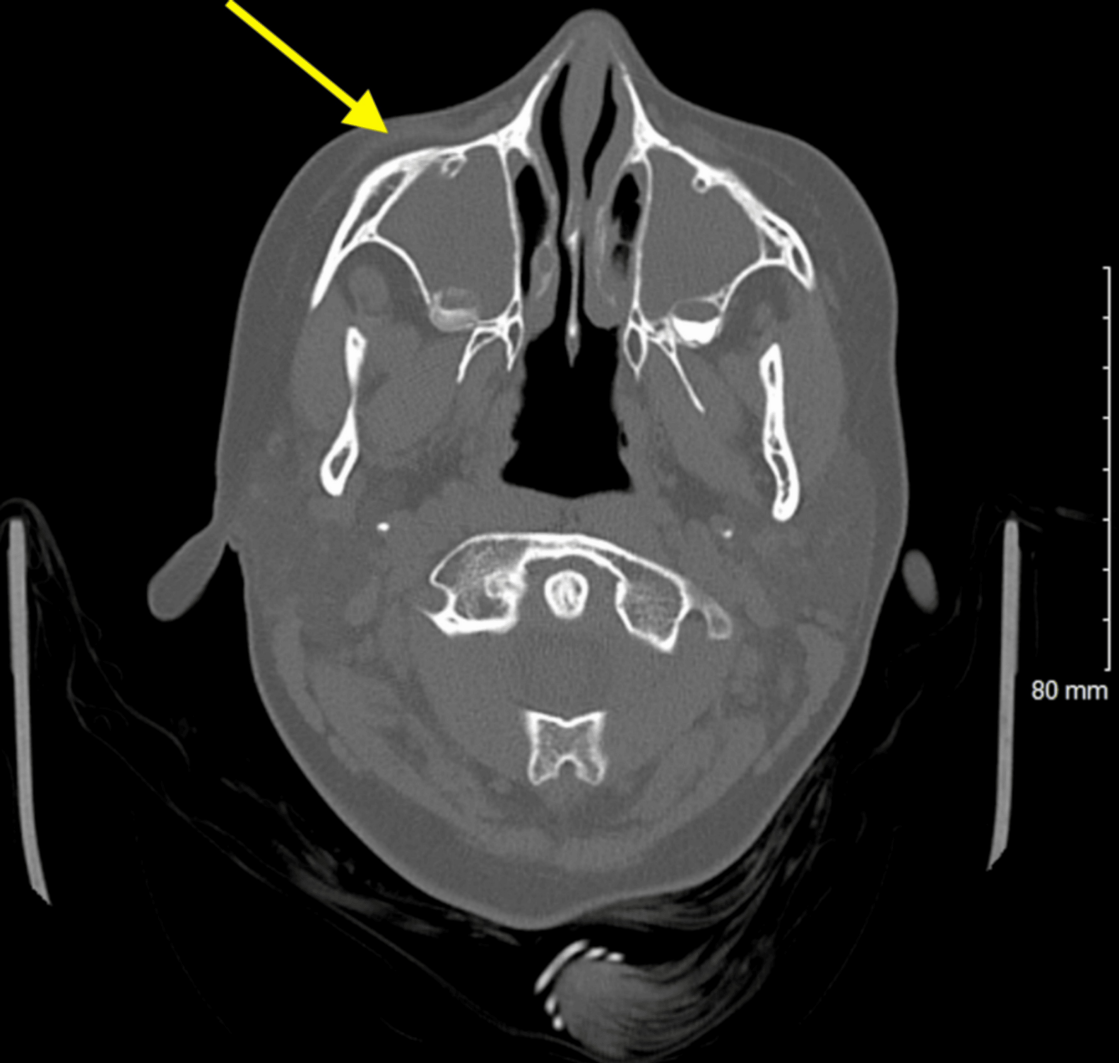
Unenhanced axial computed tomography image of the brain demonstrates extensive inflammatory changes in the sinuses bilaterally.

**Figure 4:**
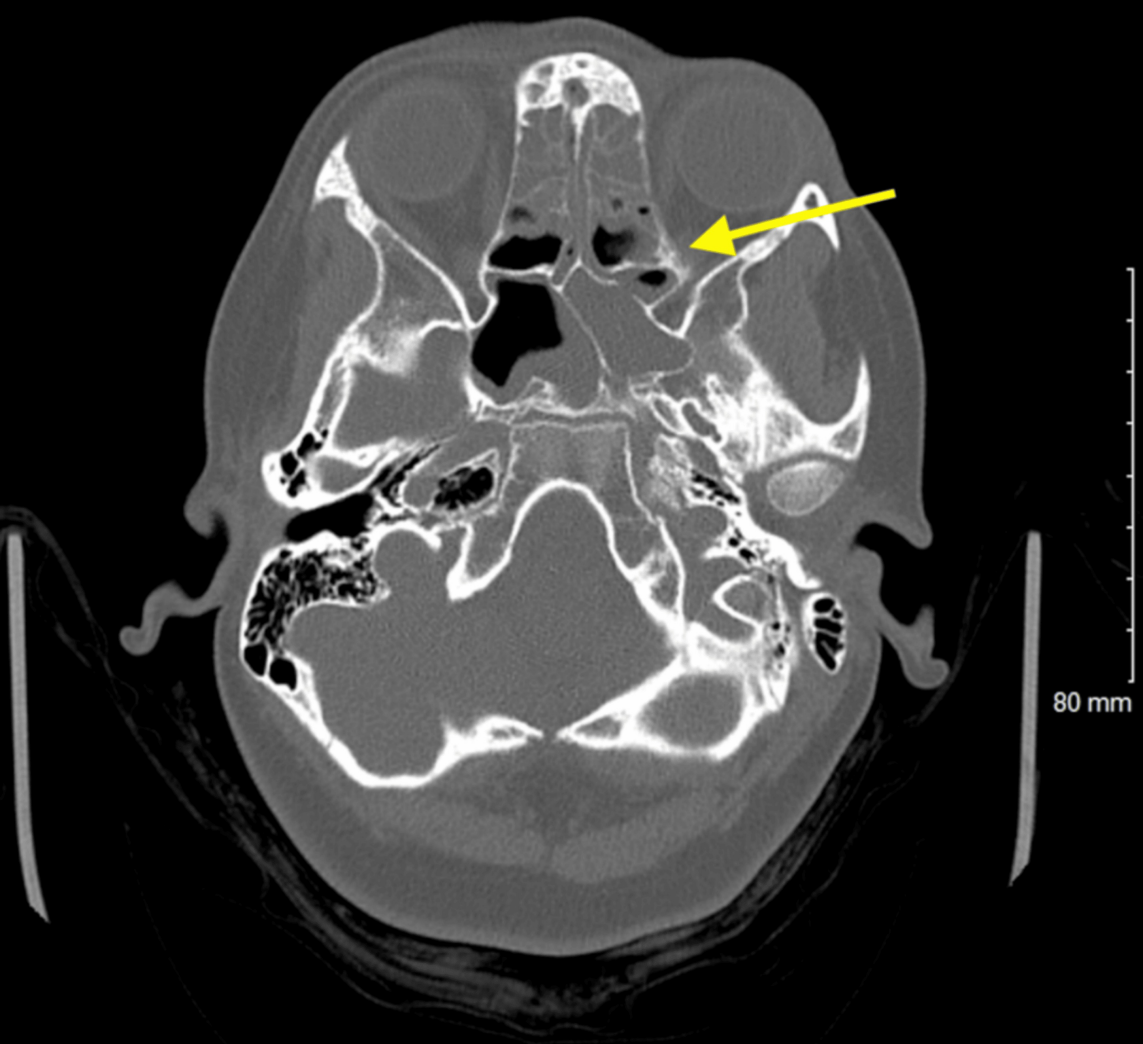
Unenhanced axial computed tomography image of the brain demonstrates extensive inflammatory changes in the sinuses bilaterally.

A computed tomography (CT) scan of the head without contrast was performed. The CT of the head (Figures 1, 2) revealed air present within the superior sagittal sinus and the extra-axial space along the frontal lobe in the midline, slightly to the right of the midline. There were inflammatory changes present throughout all of the sinuses (Figures 3, 4) with an air-fluid level in the left frontal sinus. No bony erosion was identified in this area. No abnormal extra-axial fluid collection was seen.

After receiving the CT results, vancomycin 750 mg IV, ceftriaxone 1 gram IV, and metronidazole 500 mg IV were given. The patient was transferred to a tertiary care pediatric facility for admission. Consults were placed in pediatric neurosurgery, infectious disease, and otolaryngology (ENT). 

A magnetic resonance imaging (MRI) scan of the brain with and without IV contrast and CT of the sinuses without IV contrast were obtained for further investigation. MRI of the brain showed sinusitis and a small amount of pneumocephalus which appeared similar to CT findings. No epidural abscess, subdural empyema, dural sinus thrombosis, or brain parenchymal abnormality was identified. Imaging resolution was unable to capture cribriform plates or other bony defects. CT of the sinuses without IV contrast revealed sinusitis with a small amount of pneumocephalus, unchanged from previous imaging. No obvious epidural abscess or subdural empyema was identified. Imaging was not suggestive of intracranial infection or abscess. 

The pediatric neurosurgery and otolaryngology specialists did not feel surgical intervention or sinus drainage would be warranted and conservative management was recommended. The infectious disease specialists recommended continued IV and PO antibiotics. She was later discharged with a peripherally inserted central catheter (PICC) for continued IV ceftriaxone and PO metronidazole and linezolid for two weeks to six weeks.

## Discussion

Pneumocephalus is the presence of intracranial gas, most typically air, in the intracranial space. This condition is a medical emergency, requiring immediate care and management. The term encompasses gas in any of the intracranial compartments. The most common cause of pneumocephalus is mechanical trauma. Other possible causes are instrumentation such as recent neurosurgery, external ventricular drain placement, sinus surgery, or peridural anesthesia. It is important to note that after a supratentorial craniotomy, pneumocephalus may persist in a minority of patients into the third week postoperatively but should not last beyond this time [6]. Further identified causes include barotrauma, otogenic pneumocephalus, meningitis involving a gas-forming organism [7], and sneezing [8,9]. It may also occur spontaneously [10].

CT scanning of the head without contrast is the gold standard imaging modality in diagnosing pneumocephalus. This study can detect even 0.55mL of intracranial air. MRI of the brain is less sensitive than CT in diagnosing pneumocephalus.

Pneumocephalus is usually asymptomatic in mild cases and can be classified into simple or tension types based on severity and progression. Symptoms of pneumocephalus vary based on the amount of air present and its location within the cranial cavity. Larger amounts of intracranial air can cause headaches, dizziness, nausea, and neurological deficits. A small number of patients with pneumocephalus describe “bruit hydroaerique,” which is a splashing noise or gurgling sensation on head movement and can even be audible via the use of a stethoscope to an examiner [11,12].

Treatment depends upon the underlying cause and prognosis is generally good for patients with simple pneumocephalus. Given that the majority of cases are an expected finding in the post-craniotomy patient, often no treatment is necessary as the gas will gradually be reabsorbed. Conservative treatment measures include bed rest, placing the patient in the Fowler position at 30°, avoiding Valsalva maneuvers (nose blowing, coughing, and sneezing), administering analgesic and antipyretic medications to prevent hyperthermia, osmotic diuretics (if indicated), oxygen therapy at 5L/min or 100% non-rebreather mask, and hyperbaric oxygen therapy. Positive pressure ventilation should be avoided [13].

In a historic case series, the rate of pneumocephalus in the setting of infection without a bony defect of the cranium accounted for only 1% of cases [5]. Of those cases, the majority are otogenic in nature [5]. There are case reports of pneumocephalus resulting from bone defects that are not otogenic in nature. In this group, the most frequent were defects of the sphenoid sinuses and cribriform plate [14,15]. This leads to communication between the inside of the skull and the intracranial compartment, which allows air to penetrate the cranium. Symptoms may be preceded by a Valsalva maneuver (e.g., blowing the nose, sneezing) [10,16,17], though in a significant number of cases, the triggers remain unknown [10,18]. Headache is the most commonly described symptom, along with fever [14,19]. Germiller et al. found in their pediatric study of intracranial complications of sinusitis that over half of patients had facial mass or swelling at presentation [19]. For our patient, parents had reported periorbital swelling the day prior to presentation. In that same study, 58% lacked any central nervous system symptoms at presentation and 59% had a normal neurologic examination [19]. Due to these findings, it is recommended that clinicians should maintain a high index of suspicion in pediatric patients, as they often present with vague signs and symptoms [19]. Medical management of intracranial complications of sinusitis requires aggressive IV antibiotic therapy with agents that will penetrate the cerebrospinal fluid (CSF) [19]. Early consultation with several specialists is critical, including infectious disease, neurosurgery, and otolaryngology. Nash et al. presented the case of a 27-year-old woman diagnosed with intraparenchymal pneumocephalus which was localized in the right frontal lobe, due to a cribriform plate defect [20].

In our patient, it was suspected that her preceding bouts of sinusitis had led to a bony defect which was too small to characterize on imaging. Given that the location of air in the right frontal lobe has been documented in the past to be associated with a cribriform plate defect, we suspect our patient may have had a similar defect. The patient had reported a recent nose blowing due to sinus and nasal congestion. This Valsalva maneuver was thought to have resulted in a sudden pressure increase in the sinuses, allowing air to be drawn into the cranium through the bony defect and resulting in pneumocephalus.

## Conclusions

Pneumocephalus is a potentially life-threatening emergency. It requires prompt diagnosis and treatment directed toward the underlying cause. Presentations and symptoms will vary depending on the amount of intracranial air. Because of the variance in presentation, clinicians should maintain a high index of suspicion in pediatric patients, especially those with preceding sinus history as they often present with vague signs and symptoms. Most cases of pneumocephalus are traumatic or post-surgical in nature and respond to conservative treatment. In the case of a known or suspected infectious etiology, broad-spectrum IV antibiotic coverage with CSF penetration should be initiated and early consultation with infectious disease, neurosurgery, and otolaryngology is essential.
